# A Multifaceted Independent Performance Analysis of Facial Subspace Recognition Algorithms

**DOI:** 10.1371/journal.pone.0056510

**Published:** 2013-02-25

**Authors:** Usama Ijaz Bajwa, Imtiaz Ahmad Taj, Muhammad Waqas Anwar, Xuan Wang

**Affiliations:** 1 Department of Computer Science, COMSATS Institute of Information Technology, Abbottabad, Pakistan; 2 Vision and Pattern Recognition Systems Research Group, Muhammad Ali Jinnah University, Islamabad, Pakistan; 3 Center for Advanced Studies in Engineering (CASE), Islamabad, Pakistan; 4 Department of Computer Science & Technology, Harbin Institute of Technology, Shenzen Graduate School, Shenzen, China; University of Adelaide, Australia

## Abstract

Face recognition has emerged as the fastest growing biometric technology and has expanded a lot in the last few years. Many new algorithms and commercial systems have been proposed and developed. Most of them use Principal Component Analysis (PCA) as a base for their techniques. Different and even conflicting results have been reported by researchers comparing these algorithms. The purpose of this study is to have an independent comparative analysis considering both performance and computational complexity of six appearance based face recognition algorithms namely PCA, 2DPCA, A2DPCA, (2D)^2^PCA, LPP and 2DLPP under equal working conditions. This study was motivated due to the lack of unbiased comprehensive comparative analysis of some recent subspace methods with diverse distance metric combinations. For comparison with other studies, FERET, ORL and YALE databases have been used with evaluation criteria as of FERET evaluations which closely simulate real life scenarios. A comparison of results with previous studies is performed and anomalies are reported. An important contribution of this study is that it presents the suitable performance conditions for each of the algorithms under consideration.

## Introduction

Due to growing requirements of non-invasive recognition systems, Face Recognition has recently become a very popular area of research. A variety of algorithms for face recognition have been proposed and a few evaluation methodologies have also been used to evaluate these algorithms. However, current systems still need to be improved to be practically implementable in real life problems.

A recent comprehensive study [1], categorizes and lists the popular face recognition algorithms and databases. This study has categorized face recognition algorithms into five categories namely linear and non-linear projection methods, neural network based methods (another non-linear solution), Gabor filter and wavelets based methods, fractal based methods and lastly thermal and hyperspectral methods. However [2], in their study grouped the approaches of face recognition into two broad categories, namely appearance based and feature based. Although many feature based algorithms have been proposed [3]–[6] etc, they have limitations due to their heavy dependency on feature detection methods, which are mostly prone to error. Moreover, due to inherent variability of facial structure, the feature metrics are not reliable under varying expressions and temporal changes. Appearance based face recognition algorithms, on the other hand, despite being dependent on primitive pixel values are still considered to be a better choice [2]. Among the appearance based methods, the so called subspace methods which rely on the dimensionality reduction of face space while preserving the most relevant information are the most famous.

Another recent and robust face recognition algorithm [7] based on sparse representation of facial data has achieved great fame due to better performance. In this algorithm however learning stage is virtually non-existent and all the training data is used directly in the classification stage. In the classification stage, an objective function is minimized using the test image and all the training data and classification is based on the solution vector of this optimization problem. Therefore using this algorithm, precise choice of feature space is no more a critical matter, which is the focal point of our study. The sparse approach for face recognition is obviously computationally intensive at the classification stage especially for large scale systems. Therefore sparse approach does not come under the scope of our study where the feature extraction approaches and choice of distance metrics are focused, emphasizing on computational efficiency especially in the classification stage.

A large variety of subspace face recognition algorithms have been proposed in different studies including some recently proposed methods. An interesting observation about these studies is that each proposed method claims to give the best recognition rates. However, since every study use their own datasets and implementation parameters specifically designed to highlight their own performance, individual performance analysis are misleading. Therefore it is of great significance that an unbiased comparative analysis of these algorithms under equal and testing working conditions is done. The evaluation methodology is therefore very important and it should be designed to simulate the real world problems. It is very difficult to find such comprehensive evaluation methodologies in the literature, the only exemplary evaluation method being that of the FERET evaluations run by National Institute of Standards and Technology (NIST) [8].

A comparative analysis should be fair not only in terms of the databases and testing methodology but also in terms of operating conditions such as trying a complete group of classifiers for all candidate subspace methods. Trying different classifiers/distance metrics may actually bring out the strengths of a subspace projection algorithm, which may not be visible on a single metric. However, very few studies been directed towards comparative analysis of subspace based algorithms and even fewer studied the effect of different distance metrics on the algorithms for their comparison.

One of the early studies [9] used FERET [10] database with 425 gallery and training images of their own choice. The study [11] also used FERET database, but the system was trained on 675 images belonging to 225 classes and tested on 640 images belonging to 160 classes. Another study [12] did follow the FERET evaluation methodology, but just compared two algorithms PCA [13] and ICA [14] and three distance metrics not including Mahalanobis based distance metrics. The study [15] compared different hybrid approaches and used FERET database with their own selection of 762 images belonging to 254 classes for training and 254 images for testing purposes. Another study [16], which represents a face image as a spatial arrangement of image patches and derives optimal Voltera Kernels compared the performance of their proposed method with traditional and state of the art algorithms on three databases. Recently, an improved version of the said method was also introduced which employs a modern stochastic optimization algorithm [17]. A comparatively larger and latest study [18], compared three algorithms PCA, ICA and LDA [19] on the FERET database. They adopted the FERET recommendations by using the recommended test and gallery sets but they used their own training set of 675 images belonging to 225 classes.

This study was motivated due to the lack of comprehensive comparative study of many subspace methods with many distance metric combinations. Comparative studies found in the literature are limited in their scope in terms of the testing methodology and the number of test vectors and test parameters being used in the analysis. This study, unlike earlier studies, compares different algorithms based on theoretical aspects, such as resultant data structure sizes and algorithm complexity, as well as recognition rates on different facial databases. Three different databases have been used, namely FERET, YALE [19] and ORL [20]. Due to obvious reasons, the evaluation criteria chosen is the same as of FERET evaluation tests and almost similar to that for YALE and ORL. The evaluation methodology also ensures that every candidate subspace algorithm is operated at its optimal performance by using various distance metrics against each algorithm and choosing the best one.

Six subspace projection methods have been included in the comparison, which are evaluated using four distance metrics. These methods include, 1DPCA [13], 2DPCA [21], A2DPCA and (2D)^2^PCA [22], LPP [23] and 2DLPP [24]. Selection of these six algorithms is due to their efficiency and the property of being scalable to large databases. ICA has not been included in the study because it has already been thoroughly investigated in other comparative studies. LDA has also not been included because it needs class information during training and does not suit generalized evaluation methodology adopted here. The evaluation of 2DPCA and LPP is interesting due to the fact that the original studies did not use FERET database and hence missed an important facial database to present their results. The results of 2DLPP were also shown on limited test vectors using subsets of FERET database or using different training or testing sets than the ones specified by FERET evaluations.

The rest of the paper is organized as follows: Section 2 describes the subspace algorithms under consideration, Section 3 explains the evaluation methodology followed, Section 4 presents the results and related discussion and section 5 concludes the whole study and proposes future work to be done.

## Subspace Algorithms to be Evaluated

**Figure 1:**
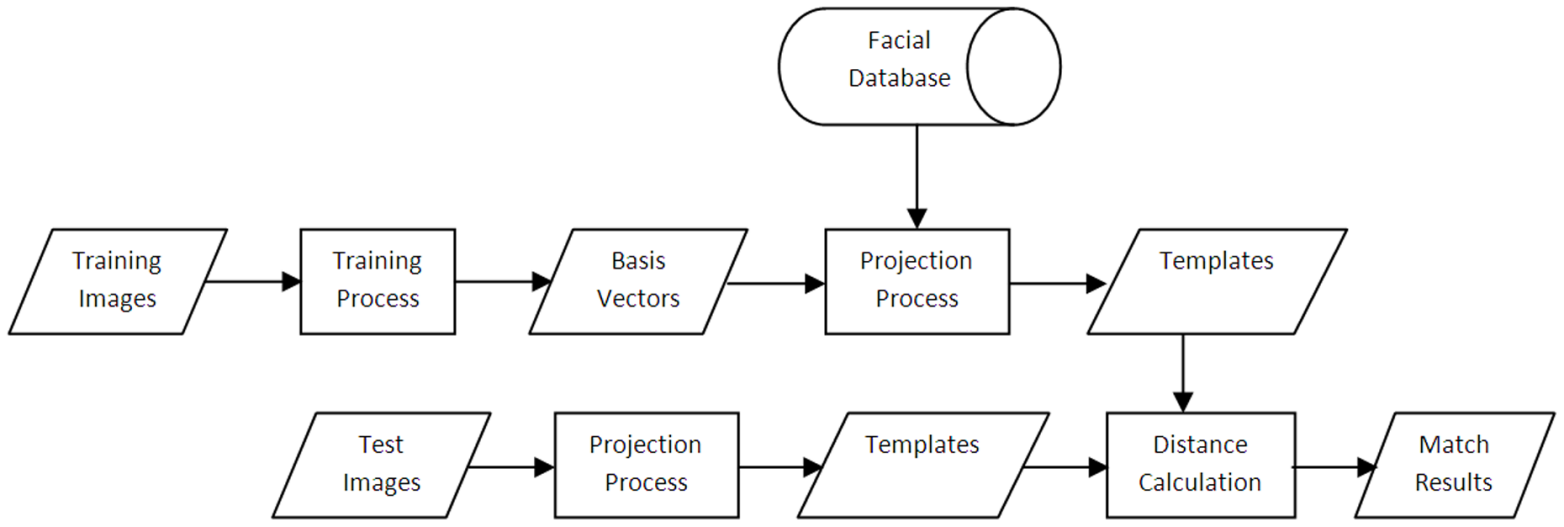
Face Recognition Process.

Three basic steps of recognition system are training, projection, and recognition. During the training phase, the basis vectors of the subspace for each algorithm are calculated and saved. During projection, these basis vectors are loaded and then all the database images are projected onto these basis vectors, which convert them to the lower dimensional subspace. These projected images are saved as templates to be later used in distance calculation in the recognition phase. The whole process is shown in figure 1.

Since all the algorithms used in this study are well known, they will be described briefly for the sake of completeness. These algorithms are referred to as subspace methods because they project the images to lower dimensional space to perform recognition task which is not computationally feasible to be done in high dimensional space. These algorithms retain maximum possible discriminative information while converting the images to lower dimensional space. Their property of retaining maximum discriminative information is what prioritizes them over each other.

Table 1 summarizes the basic implementation parameters for all the algorithms discussed in this study, listing the matrix dimensions and the time/computational and space complexity. Therefore this table is used as a reference for the purpose of comparison based on memory and computational requirements besides the recognition rates mentioned in results section. The algorithm complexity section of the table has been extended from [24]. The size of images is *mxn* (*m* is the number of rows and *n* is the number of columns of an image), *N* is the number of training images and *M* is the number of images projected on the projection matrix resulting from training. Number of retained basis vectors is *d* (*d_1_* and *d_2_* in case of 2D^2^PCA algorithm) which determines the number of dimensions preserved. A summarized description of the six subspace projection algorithms is given in the following subsections.

**Table 1:** Matrix Dimensions, Time/Computational Complexity and Space Complexity for Subspace Algorithms.

	Matrix Dimensions/Size				Algorithm Complexity		
Algorithm	Training Images	Covariance Matrix	Projection Matrix	Projected Images/Templates	Training Time	Testing Time	Memory Space
**PCA**	mn x N	NxN	mn x d	d x M	O(m^2^n^2^d)	O(MNd)	O(m^2^n^2^)
**2DPCA**	m x n x N	n x n	n x d	m x d x M	O(n^2^d)	O(mMNd)	O(n^2^)
**A2DPCA**	m x n x N	m x m	m x d	n x d x M	O(m^2^d)	O(nMNd)	O(m^2^)
**2D^2^PCA**	m x n x N	n x n & m x m	n x d_1_& m x d_2_	d_2_ x d_1_ x M	O(n^2^d_1_+ m^2^d_2_)	O(d_2_MNd_1_)	O(m^2^+ n^2^)
							
**LPP**	mn x N	NxN (at PCA step)	mn x d_LPP_	d_LPP_ x M	O(m^2^n^2^d + mnN^2^)	O(MNd)	O(m^2^n^2^)
**2DLPP**	m x n x N	N/A	n x d	m x d x M	O(n^2^d + mnN^2^)	O(mMNd)	O(n^2^)

### 1. Eigenfaces (PCA/1DPCA)

**Figure 2:**
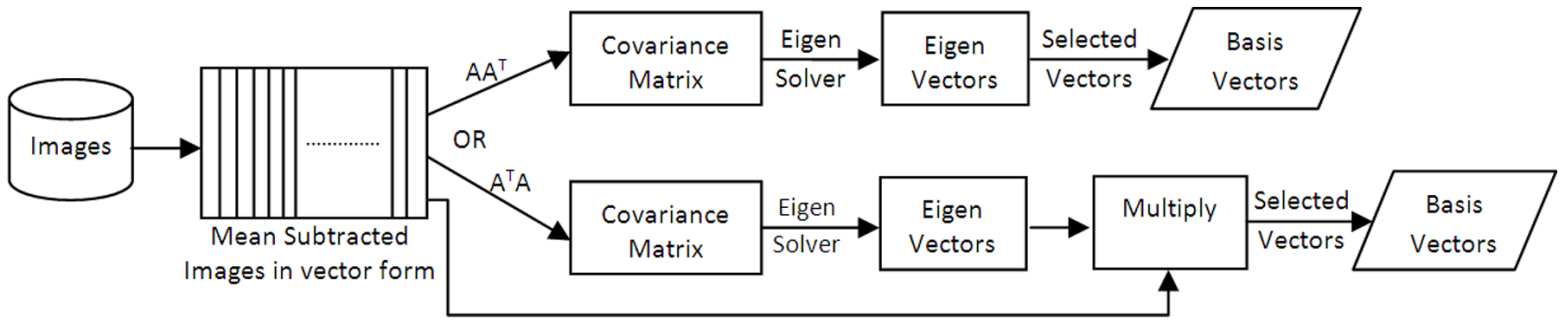
PCA Training Process.

Principal Component Analysis (PCA) [13] relies on a set of basis vectors which correspond to maximum variance direction of the image data. As suggested by the study, the calculation of covariance matrix is reduced by calculating the *A^T^A* matrix as the covariance matrix rather than *AA^T^* as in equation 1, where *A* is the matrix containing all the image vectors. This reduction is compensated by later multiplying the images *A* with Eigen vectors of the *A^T^A* matrix as in equation 2. This finally results into Eigenfaces, which are the basis vectors and serve as the projection matrix. This training process of PCA is shown in Figure 2, where both options of direct covariance and indirect covariance methods are shown. These basis vectors are normalized before further use and the reason is discussed in section 3.2.3. A specific number of vectors are retained corresponding to the same number of highest Eigen values of the covariance matrix. The images are then projected onto these retained basis vectors to find a set of weights (templates) describing the contribution of each basis vector in image reconstruction.

Suppose there are *N* images of size *mxn*, reshaping each image to a vector will result into a matrix *A* of size *mnxN* containing all images in the form of vectors of length *mn*. The image covariance matrix *G* of the size *NxN* is calculated as shown in equation 1.
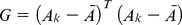
(1)Where *A_k_* is the *k’th* image in vector form from matrix 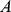 and 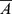 is the average image. Solving Eigen values of *G* will result into *NxN* Eigen vectors. Multiplying the images with these Eigen vectors will result in the basis vectors *B*, which is represented by equation 2.



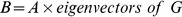
(2)These *mn* dimensional basis vectors *B* are then normalized. Corresponding to the *d* largest Eigen values calculated above, *d* vectors out of *N* vectors of *B* are chosen. These chosen vectors, also called Eigen faces, form the projection matrix *P* which is of size *mnxd*.

In the projection phase the desired *M* number of images vectors *E* are projected onto this projection matrix to get the templates which are of the of size *dxM* as shown in equation 3

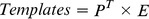
(3)


### 2. Two Dimensional PCA & Alternative Two Dimensional PCA (2DPCA & A2DPCA)

In 2-D PCA [21] and Alternative 2-D PCA [22], image covariance matrix is calculated directly using the 2D images. As evident from table 1, size of covariance matrix for 2DPCA is smaller than the one for PCA. Though 2DPCA is computationally better than PCA in training phase, it requires more storage space for the templates and more computations in the recognition phase as compared to PCA. Since 2DPCA works along the row direction of images, it preserves the variation between rows of an image taken as feature vectors. In A2DPCA however, the variation between columns of an image taken as feature vectors are preserved.

Suppose there are *N* images of size *mxn*. The image covariance Matrix *G* of size *nxn* is calculated using equation 4,
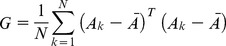
(4)Where *A_k_* is the *k’th* image and 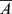 is the average image. The next step is solving for *d* Eigen vectors of *G* corresponding to the largest *d* Eigen values. These chosen *d* Eigen vectors compose the projection matrix *P* of size *nxd*. During projection, the images are projected one by one on this projection matrix. If there are a total of *M* images to be projected, the resulting templates will be of size *m x d x M*.

In case of A2DPCA, it works in column direction of images; therefore the difference is in calculating the image covariance matrix *G,* now with size *mxm* as shown in equation 5.
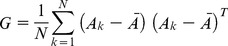
(5)


Therefore for A2DPCA, the projection matrix *P* will be of size mxd and the resulting templates will be of size *n x d x M*.

### 3. 2-Directional 2-Dimensional PCA ((2D)^2^PCA)

As discussed above, 2DPCA and A2DPCA preserve the variance between rows and between columns of the image respectively. The disadvantage of 2DPCA and A2DPCA is that they have a relatively bigger template size as compared to that of PCA which is evident from table 1. Template size is an important factor in characterizing the storage and computational requirements at the recognition stage. (2D)^2^PCA [22] possesses a comparatively reduced template size. In (2D)^2^PCA, the images are projected simultaneously on both row based and column based optimal matrices.

Suppose there are *N* images of size *mxn*. For (2D)^2^PCA algorithm, two covariance matrices are needed to be calculated using equation 4 and 5. One is *G_1_* of size *nxn* and the other is *G_2_* of size *mxm*. Solving for *d_1_* Eigen vectors of *G_1_* and *d_2_* Eigen vectors of *G_2_* corresponding to the *d_1_* and *d_2_* largest Eigen values respectively, two projection Matrices *P_1_* of size *nxd_1_* and *P_2_* of size *mxd_2_* are achieved.

In the projection phase the two dimensional images *E_k_* are simultaneously multiplied with both projection matrices to transform them into the new lower dimensional space as shown in equation 6. The projected size is *d_2_ x d_1_ x M*, where *M* is the number of images to be projected.
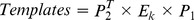
(6)


### 4. Laplacianfaces (LPP)

Laplacianfaces (LPP) algorithm [23] is a subspace algorithm that applies dimensionality reduction while preserving the locality information of feature space. In LPP, each input face is first projected to PCA subspace and stored as a single vector in the data matrix *A*, which acts as an input to LPP. An adjacency matrix *S* of a fully connected graph is computed, where each node represents an image *A_k_* in the face-space. Weights are assigned to the edges in the connected graph on the basis of a fixed neighborhood of *K* samples. The weight of an edge is determined by the measure of closeness of nodes.
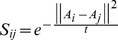
(7)


In equation 7, *S_ij_* represents the weight of the edge connecting node *A_i_* and *A_j_* in the adjacency graph *S*. The parameter *t* in the above equation controls the spread of the neighborhood and that encompasses *K* nearest neighbors. In this study the parameter *t* is computed using equation 8.
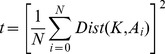
(8)Where, *N* is the number of training set images, *Dist* is the distance matrix in which each column contains sorted distances of an image with all images. The matrix *A* contains all input images projected into PCA subspace as vectors; *A_i_* represents a particular image in the matrix *A* on the index *i*. A diagonal matrix, *D* is computed by adding all elements in a row of the matrix *S*, and placing the sum in the diagonal elements. Laplacian Matrix *L* is calculated by subtracting adjacency matrix *S* from diagonal matrix *D*. An optimized embedding is then computed by solving the generalized Eigen problem given in equation 9 that yields the Eigen values *λ* and Eigen vectors *w*. These Eigen vectors are used as subspace basis vectors, referred to as *P_LPP_* in equation 10.



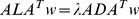
(9)These *d* vectors are chosen corresponding to the *d* smallest Eigen values, referred as *d_LPP_*. The complete projection matrix *P* is shown in equation 10, where
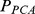
are the subspace basis vectors of PCA subspace.
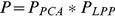
(10)


In the projection phase, using equation 3, the desired *M* number of images *E* are projected to get the templates in the Laplacian subspace which are of the size *d_LPP_ x M*.

### 5. Two Dimensional Laplacianfaces (2DLPP)

Two Dimensional Laplacianfaces (2DLPP) [24] is a recently proposed method for face recognition. In 2DLPP the 2D images are used directly without converting into vectors first. The adjacency matrix *S*, neighborhood spread *t*, diagonal matrix *D* & Laplacian matrix *L* are calculated as in LPP method. For computing the optimized embedding, the generalized Eigen problem of equation 11 is solved which is different from the one used in LPP method. The reasoning for such change is given in [24].
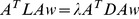
(11)


The *d* selected Eigen vectors corresponding to the smallest *d* Eigen values constitute the projection matrix *P*. An image in the face-space can thus be projected onto the 2DLPP subspace.

## Evaluation Methodology

The evaluation methodology followed in this study is explained by addressing the training and projection method and the testing variables used in the evaluation. A MATLAB based evaluation platform that is constructed as a result of this study is also described.

### 1. Basic Modules of Evaluation System

**Figure 3:**
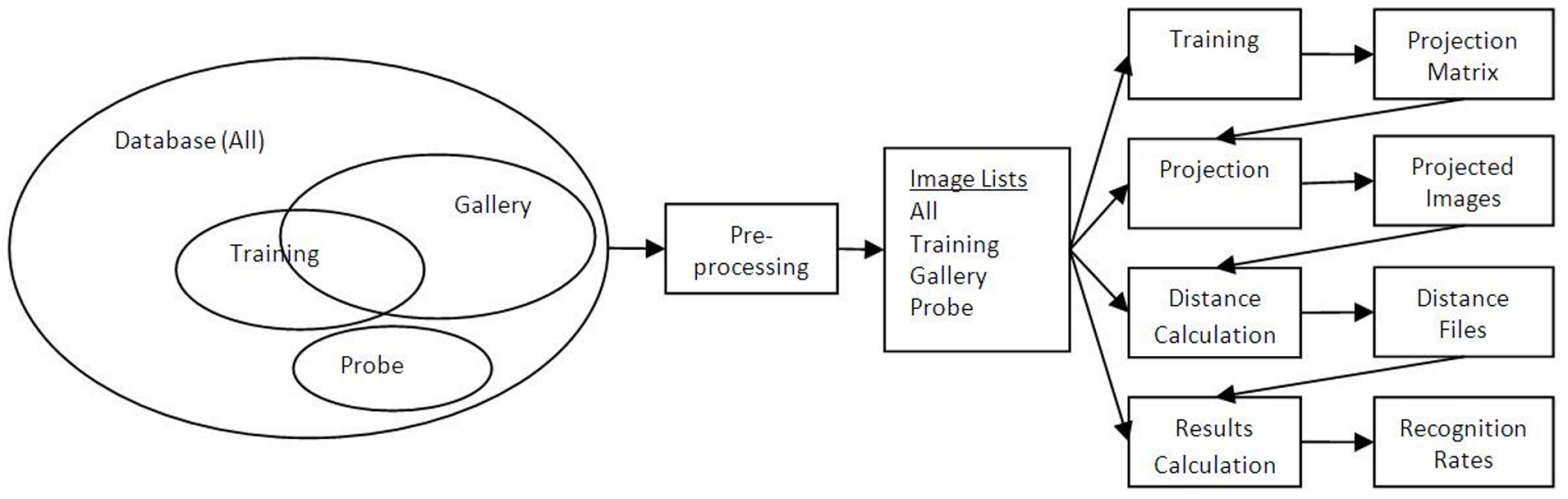
Basic Modules of Evaluation Methodology.

Four basic modules of the evaluation methodology include Training, Projection, Distance Calculation and Result Calculation as shown in figure 3. To ensure a uniform evaluation for all methods, the images used for training and testing are predetermined and stored in the form of image lists. For example the image list “all_feret” contains the names of all the images for the FERET database. “train_feret” is the image list containing training images from FERET database. Similarly four probe image lists for FERET and one each for YALE and ORL contain the names of images to be used for testing the system. The “gallery” list contains the names of the images against which the probe set images are to be compared. Given a query face image, the probe, the system has to find most similar out of the known faces in the gallery, while the system has been trained on the training set that is a small subset of the database.

For FERET, the training, gallery and probe sets are already defined by FERET evaluation tests [8]. Similar arrangements are done for image lists for YALE and ORL. More details regarding the structure of database and image sets have been given in Section 3.2.1.

Prior to training, FERET and Yale images have been pre-processed by first alignment using eye coordinates to compensate head tilt, then illumination adjustment using histogram equalization, then cropping using an elliptic mask so that only face is visible, and finally resizing to 150×130 pixels. ORL isn’t processed because it has minimal background variation and limited head tilt. In case of FERET the eye coordinate file is supplied along with the database. For YALE database, eye coordinates are manually selected and a similar eye-coordinates file is maintained.

During the training phase, the projection matrix is trained using the images from the training image list of a particular database by the projection algorithm to be evaluated. The size of the projection matrix is determined by the retained percentage of basis vectors.

In the projection phase, the images listed in the “all” image list of the specific database are projected onto the face subspace using the projection matrix and saved as the output of this phase. The training and projection operation along with the rest of operations is shown in figure 3.

In distance calculation phase, the distances between a projected probe image and all other projected images in the gallery are calculated and written in a file named after the name of the projected image. The same is repeated for every projected image against the distance metric of our choice. These distance files are later used in the result calculation phase.

In the result calculation phase, the gallery and probe image lists are read and the distance file for each probe image is loaded to check if the closest match is among the images named in gallery list. Here the match scores are calculated against each Rank. Rank 1 means the first match and Rank 50 means 50^th^ match. The results are calculated for all the probe sets and saved.

### 2. Testing Variables

Table 2 summarizes all the testing variables used in the evaluation process.

**Table 2:** Testing Variables.

Databases	FERET				ORL		YALE	
Probe Sets	fafb	fafc	dup1	dup2	probe		probe	
Algorithms	PCA		2DPCA		A2DPCA	(2D)^2^PCA	LPP	2DLPP
Distance Metrics	Euclidean				Cosine	Mahalanobis	Mahalanobis Cosine	

#### 2.1. Databases

Three databases are selected for our comparative study, namely FERET, YALE and ORL. The description and reasons for choosing these databases is given in the following paragraphs.

FERET database has been extensively used by FERET evaluation tests, face recognition vendor tests (FRVT) and by many researchers for different research algorithms as well as commercial face recognition systems [8]. FERET has been chosen to test the performance of the algorithm combinations under conditions where there is a variation in facial expressions, lighting conditions and temporal changes. The experiments here use the standard image subsets as in FERET evaluation test. These image subsets include an image set for training which consists of 501 images of randomly selected 428 subjects and the images per subject range from minimum 1 to maximum 3. A gallery set of 1196 images and four probe sets namely fafb, fafc, dup1 & dup2 totaling 2345 images are used. The gallery set consists of one image for each of the 1196 subjects with neutral expression. The probe sets are used to assess the performance of the algorithm against several conditions. For evaluation against change in expression, the probe set “fafb” is used. Similarly for evaluation against different illumination conditions, the probe set “fafc” is used. For evaluation against temporal/aging changes the dup1 and dup2 probe sets are used. It is necessary to mention that among the total 3368 frontal images used in this study, there are subjects having images with and without glasses. The details of number of images per set are shown in table 3.

**Table 3:** Databases, training and test set details.

Evaluation Against	Probe Set Names	No. of Gallery Images	No. of Images in Probe Set
FERET Database			
Expression	Fafb	1196	1195
Illumination	fafc	1196	194
Aging	dup 1	1196	722
Aging	dup2	1196	234
**No. of images in the training set**		501	
**No. of Subjects**		1196	
**No. of images per subject**		1 to 25	
**No. of total images**		3368	
ORL Database			
General Evaluation	probe	40	200
**No. of images in the training set**		200	
**No. of Subjects**		40	
**No. of images per subject**		10	
**No. of total images**		400	
YALE Database			
General Evaluation	probe	15	75
**No. of images in the training set**		90	
**No. of Subjects**		15	
**No. of images per subject**		11	
**No. of total images**		165	

The ORL database [20] is one of the famous older databases. The reason why it is chosen is because it has been used by the authors of the algorithms under discussion in our study. There are 10 different images for each of 40 distinct subjects hence totaling to 400. For some of the subjects, the images were taken at different times and slight variations in illumination, facial expression, facial detail, head tilt, pose angle and scale of face area in an image are present. All the images were taken with constant dark background and most of them are frontal. The training set is chosen to be the first five images for every subject which becomes 200 images in total. One frontal image with neutral expression is manually selected for each of the 40 subjects to be included in the gallery set. Only one probe set is used which consists of the last 5 images for every subject which totals to 200 images. This training and probe set combination has already been used by 2DPCA and (2D)^2^PCA authors. The database and its relevant details are summarized in table 3.

The YALE database [19] consists of 165 images belonging to 15 subjects thus having 11 images per subject. Images belonging to this database possess 3 variations in lighting condition, 6 variations in facial expression, with glasses and without glasses. This makes one image per variation for each subject. Our experiments on this database use the same testing criteria as that of [23]. Training set is constructed by randomly picking six images per subject so that all 11 variations get the chance of being part of training set, therefore 90 images in total are used for training. The rest of the database is considered to be the only probe set having a total of 75 images. The gallery comprises of one image with normal facial expression for each subject i.e. 15 images. The specifics of image sets are given in table 3.

#### 2.2. Distance Metrics

Four distance metrics are chosen including Euclidean (L2) and Cosine for the image space and their counter parts in Mahalanobis space, Mahalanobis (L2) & Mahalanobis Cosine. These metrics are referred throughout the study as Euc, Cos, Maha and MahCos, respectively. The Mahalanobis space based distance metrics are applied by transforming the templates from image space to Mahalanobis space. For each vector pair u and v in image space the transformed vector pair m and n in Mahalanobis space is given as in equation 12

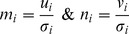
(12)Here *σ_i_* is the standard deviation of the *i* ^th^ dimension. Calculation of this standard deviation/spread is discussed in section 3.2.3.

For the sake of completeness, mathematical description of each distance metric is given below.

Euclidean/(L2)/(Euc): The Euclidean/L2 distance between two vectors *u, v* in image space is calculated as in equation 13.
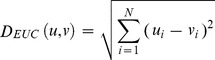
(13)


Cosine/(Cos): The Cosine distance between two vectors *u, v* in image space is calculated as in equation 14

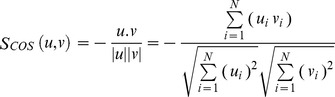
(14)


Higher similarity means higher score in this case; therefore the actual distance is calculated by subtracting the above calculated value from 1 as in equation 15.
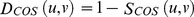
(15)


Mahalanobis/(L2)/(Maha): It is equivalent to Euclidean computed in Mahalanobis space. The Mahalanobis/(L2) distance between two vectors *u, v* in image space is calculated by equation 16.
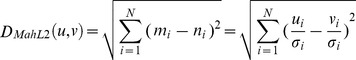
(16)


Mahalanobis Cosine/(MahCos): It is equivalent to Cosine computed in Mahalanobis space. The Mahalanobis Cosine distance between two vectors *u, v* in image space is calculated as in equation 17.
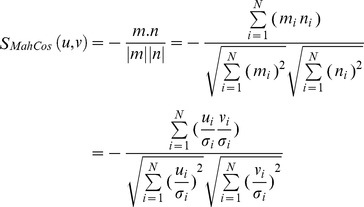
(17)


Similar to Mahalanobis, the actual distance is calculated by subtracting the above calculated value from 1 as in equation 18.
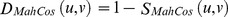
(18)


#### 2.3. Computing the data spread for Mahalanobis space transformation

As discussed in section 3.2.2, it is important to compute the standard deviation/spread to be used in calculating Mahalanobis space based distance metrics. The variance of the face data along its principal component directions is determined by the Eigen values of the image covariance matrix along all the dimensions. Therefore the spread in a specific dimension will be the square root of the Eigen value corresponding to that dimension. For PCA based algorithms, the Eigen values of the initial covariance matrix can be used as the spread at later stage to calculate Mahalanobis based distance metrics but for Laplacianfaces the Eigen values of initial covariance doesn’t represent the actual spread of Laplacian projected images. Therefore the Eigen values of the covariance matrix (of projected images) have to be calculated, to finally get to the spread.

It has been confirmed that the Eigen values of the initial covariance matrix and the Eigen values of the covariance matrix of projected images are same. An exception exists for 1D PCA, if the vectors of projection matrix/basis vectors are not normalized then the spread of projected images is square of the spread of training images. Therefore basis vectors in 1D PCA are normalized before further usage.

For the sake of similarity and generalization in the platform code, in case of 2D algorithms, the projected images are reshaped into vectors first. It is confirmed that it yields the same result either two dimensional projected images are used directly or if they are reshaped into vectors first.

### 3. Platform

As a part of this study, a MATLAB based platform FaceRecEval has also been implemented which serves the purpose of evaluating and comparing different algorithms. This platform is developed being inspired by the CSUFaceIdEval System [25]. The authors have already extended the CSUFaceIdEval System and have also ported the whole platform to the Windows operating system [26]. This work was done in context to the studies [27]–[28]. FaceRecEval will serve as a very useful tool for the fellow researchers who are more acquainted with MATLAB. Currently version 1 of this platform is available for free download [29].

All the main functionalities described in section 3.1 including training, projection, distance calculation and result calculation are incorporated in form of modules. The result calculation module calculates the results as described at start of this section. The reason behind projecting all the images and calculating the distances between all projected images is to accommodate any changes in gallery and probe image lists, because no rework prior to this module will be needed.

## Results and Discussions

For the sake of completion and to avoid confusion due to diversity of testing parameters, the results and discussions have been grouped based on recognition tasks, facial databases, distance metrics, algorithms, memory and computational complexities and comparison to previous work. Table 4 and 5 summarize the results for FERET, YALE and ORL databases. The recognition rates being displayed are the average of 50 ranks. The comparative recognition rates for each rank may vary a little and can be downloaded from [29], but the general trend remains the same, therefore here average recognition rates are shown. Recognition rates do vary for different percentage of retained basis vectors as it is evident from results and also supported by [28].

**Table 4:** Average Recognition Rate over 50 ranks for FERET database.

FERET Database		FAFC	% of basis vectors				FAFB	% of basis vectors				DUP1	% of basis vectors				DUP2	% of basis vectors			
Algorithm	Classifier	5	10	25	50	75	5	10	25	50	75	5	10	25	50	75	5	10	25	50	75
PCA	Cos	0.05	0.07	0.09	0.10	**0.11**	0.79	0.83	0.86	**0.87**	0.87	0.39	0.44	**0.47**	0.47	0.47	0.22	0.26	**0.28**	0.28	0.27
	Euc	0.06	0.08	0.10	**0.11**	0.11	0.79	0.83	0.86	**0.87**	0.87	0.39	0.44	**0.46**	0.46	0.46	0.22	0.26	**0.27**	0.26	0.26
	Maha	0.21	0.41	0.47	**0.48**	0.47	0.77	0.86	**0.89**	0.84	0.78	0.43	**0.51**	0.48	0.39	0.30	0.24	**0.30**	0.27	0.20	0.16
	MahCos	0.18	0.40	0.51	**0.56**	0.52	0.77	0.86	**0.92**	0.91	0.86	0.44	0.53	**0.54**	0.49	0.37	0.23	0.31	**0.33**	0.28	0.21
2DPCA	Cos	0.13	0.19	**0.26**	0.21	0.15	0.84	**0.85**	0.82	0.69	0.57	0.36	**0.39**	0.38	0.27	0.19	**0.16**	0.13	0.12	0.07	0.05
	Euc	0.08	0.10	**0.12**	0.10	0.09	0.90	**0.91**	0.91	0.89	0.87	0.49	**0.50**	0.47	0.43	0.41	**0.31**	0.29	0.25	0.21	0.18
	Maha	0.07	0.11	**0.12**	0.06	0.03	**0.90**	0.90	0.76	0.51	0.34	**0.46**	0.45	0.35	0.18	0.10	**0.27**	0.23	0.13	0.04	0.01
	MahCos	0.07	0.10	**0.15**	0.09	0.06	0.89	**0.90**	0.81	0.63	0.49	**0.47**	0.45	0.38	0.23	0.15	**0.26**	0.20	0.13	0.05	0.03
A2DPCA	Cos	0.14	0.32	**0.56**	0.47	0.38	**0.84**	0.82	0.79	0.71	0.67	0.44	**0.54**	0.53	0.44	0.37	0.28	0.42	**0.46**	0.34	0.24
	Euc	0.09	0.16	**0.19**	0.16	0.15	0.89	**0.90**	0.90	0.89	0.87	0.46	0.51	**0.52**	0.48	0.45	0.27	0.34	**0.35**	0.30	0.26
	Maha	0.13	0.25	**0.32**	0.12	0.06	0.87	**0.88**	0.80	0.51	0.31	0.45	**0.51**	0.45	0.20	0.10	0.28	**0.36**	0.32	0.08	0.01
	MahCos	0.13	0.26	**0.32**	0.13	0.06	0.87	**0.88**	0.81	0.55	0.34	0.45	**0.52**	0.46	0.23	0.11	0.28	**0.37**	0.33	0.10	0.03
(2D)2PCA	Cos	0.07	0.15	**0.28**	0.23	0.15	0.78	0.82	**0.83**	0.69	0.58	0.28	0.37	**0.39**	0.28	0.19	0.12	**0.13**	0.13	0.08	0.05
	Euc	0.05	0.09	**0.13**	0.10	0.09	0.87	0.90	**0.92**	0.89	0.87	0.43	0.48	**0.49**	0.44	0.41	0.24	**0.28**	0.26	0.21	0.19
	Maha	0.11	**0.24**	0.21	0.04	0.01	0.87	**0.88**	0.68	0.27	0.11	0.44	**0.47**	0.32	0.08	0.03	0.25	**0.28**	0.16	0.01	0.00
	MahCos	0.11	0.24	**0.34**	0.17	0.09	0.87	**0.88**	0.79	0.52	0.36	0.44	**0.48**	0.39	0.19	0.11	0.25	**0.27**	0.20	0.05	0.02
LPP	Cos	0.11	0.12	0.29	0.40	**0.45**	0.39	0.52	0.71	0.80	**0.83**	0.19	0.26	0.33	**0.38**	0.38	0.07	0.11	0.15	0.17	**0.18**
	Euc	0.13	0.12	0.26	0.32	**0.34**	0.35	0.48	0.61	0.66	**0.67**	0.17	0.23	**0.26**	0.25	0.22	0.06	0.08	0.10	0.11	**0.12**
	Maha	0.03	0.03	0.05	0.06	**0.08**	0.15	0.17	0.18	0.20	**0.22**	**0.10**	0.10	0.10	0.07	0.06	0.05	0.05	0.07	0.08	**0.10**
	MahCos	0.02	0.03	0.06	0.08	**0.09**	0.16	0.18	0.20	0.20	**0.23**	0.11	0.12	**0.14**	0.13	0.12	0.04	0.05	0.08	0.10	**0.15**
2DLPP	Cos	0.16	0.21	0.26	0.27	**0.28**	0.36	0.45	0.46	0.47	**0.48**	0.08	0.12	0.12	0.13	**0.14**	0.02	**0.03**	0.03	0.02	0.02
	Euc	0.01	**0.02**	0.02	0.02	0.02	0.21	**0.28**	0.27	0.27	0.26	0.03	**0.05**	0.05	0.05	0.05	**0.01**	0.01	0.01	0.01	0.01
	Maha	0.11	**0.16**	0.09	0.11	0.08	0.76	0.75	0.79	**0.80**	0.78	0.39	0.36	**0.43**	0.41	0.41	0.27	0.23	0.29	0.27	**0.30**
	MahCos	0.09	0.09	0.11	0.14	**0.15**	0.79	**0.82**	0.80	0.80	0.79	0.43	0.43	**0.44**	0.43	0.43	0.31	0.28	0.31	**0.32**	0.32

**Table 5:** Average Recognition Rate over 50 ranks for YALE database and ORL database.

YALE Database		% of basis vectors					ORL Database		% of basis vectors				
Algorithm	Classifier	5	10	25	50	75	Algorithm	Classifier	5	10	25	50	75
PCA	Cos	0.62	0.70	0.73	**0.74**	0.74	PCA	Cos	0.65	0.68	**0.70**	0.68	0.67
	Euc	0.59	0.68	**0.73**	0.72	0.72		Euc	0.68	0.70	**0.71**	0.70	0.69
	Maha	0.56	**0.69**	0.69	0.65	0.55		Maha	0.67	**0.69**	0.65	0.46	0.35
	MahCos	0.61	**0.73**	0.73	0.72	0.62		MahCos	0.66	**0.67**	0.64	0.49	0.39
2DPCA	Cos	**0.68**	0.66	0.61	0.59	0.57	2DPCA	Cos	**0.61**	0.60	0.54	0.47	0.42
	Euc	**0.76**	0.76	0.75	0.74	0.74		Euc	**0.76**	0.76	0.75	0.71	0.68
	Maha	**0.74**	0.73	0.64	0.62	0.59		Maha	**0.72**	0.70	0.55	0.31	0.20
	MahCos	**0.74**	0.73	0.66	0.63	0.61		MahCos	**0.70**	0.68	0.53	0.38	0.32
A2DPCA	Cos	**0.72**	0.71	0.61	0.60	0.57	A2DPCA	Cos	0.65	**0.69**	0.60	0.52	0.48
	Euc	**0.77**	0.77	0.74	0.73	0.72		Euc	0.73	**0.76**	0.74	0.71	0.69
	Maha	**0.76**	0.73	0.60	0.53	0.45		Maha	0.73	**0.75**	0.61	0.32	0.18
	MahCos	**0.75**	0.73	0.61	0.53	0.43		MahCos	0.73	**0.75**	0.61	0.46	0.34
(2D)2PCA	Cos	**0.68**	0.67	0.61	0.59	0.57	(2D)2PCA	Cos	0.55	**0.59**	0.54	0.46	0.42
	Euc	**0.77**	0.77	0.75	0.75	0.74		Euc	0.74	**0.75**	0.75	0.71	0.68
	Maha	**0.77**	0.70	0.51	0.53	0.49		Maha	**0.71**	0.69	0.42	0.13	0.08
	MahCos	**0.78**	0.71	0.55	0.56	0.54		MahCos	**0.71**	0.70	0.54	0.35	0.29
LPP	Cos	0.40	0.42	0.46	0.47	**0.48**	LPP	Cos	0.23	0.26	0.28	0.25	**0.29**
	Euc	0.25	0.45	**0.54**	0.44	0.45		Euc	0.17	0.18	0.30	0.26	**0.28**
	Maha	0.08	0.09	0.10	0.10	**0.11**		Maha	0.05	0.05	0.07	0.08	**0.10**
	MahCos	0.09	0.12	0.13	0.12	**0.14**		MahCos	0.05	0.06	0.08	0.08	**0.14**
2DLPP	Cos	0.36	0.40	0.47	**0.48**	0.48	2DLPP	Cos	0.21	0.22	0.21	0.24	**0.25**
	Euc	0.49	0.48	0.53	**0.54**	0.53		Euc	0.17	**0.20**	0.18	0.16	0.17
	Maha	**0.70**	0.69	0.69	0.68	0.68		Maha	0.58	0.54	0.56	0.64	**0.67**
	MahCos	0.72	0.73	**0.75**	0.74	0.72		MahCos	0.58	0.63	0.67	0.67	**0.67**

### 1. Based on Recognition Tasks

#### 1.1. Illumination Task

Starting with algorithm performance against illumination variations, it can be noted from table 4 that the recognition rates are generally lower against FAFC probe set. It is because the Eigen vectors corresponding to highest Eigen values were not dropped for the sake of similarity and generalization. For PCA based algorithms the top Eigen vectors encode most of the illumination information. Removing few of them, depending on the count of training images, might improve results of some algorithms against this task.

Two dimensional PCA algorithms perform relatively better when 25% of basis vectors are retained. LPP along with simple Cosine and Euclidean distance metrics achieves good recognition rates against this task but it is while retaining the highest percentage of basis vectors. PCA along with Mahalanobis distance variants generally perform the best for different percentages of retained basis vectors against this task. The best performing algorithms for this task are PCA-MahCos, with 50% retained basis vectors, and A2DPCA-Cos, with 25% retained basis vectors.

#### 1.2. Expression Task

The FAFB set is used to evaluate performance of an algorithm against change in expression. This is the easiest task with highest recognition rates as evident from table 4. All PCA based algorithms with Euclidean distance metric perform equally well and generally have the best recognition rates. The best performers generally for this task are PCA-MahCos with 25% retained basis vectors and 2D^2^PCA-Euc with 25% retained basis vectors. No direct conclusion can be made about the most suitable algorithm for such tasks. But the algorithms that result into the smallest template size, i.e. PCA-MahCos and 2D^2^PCA-Euc may be the best choices.

#### 1.3. Aging Task

Dup 1 and Dup 2 are the two sets provided to test the performance of algorithms against temporal changes. Dup 2 being the harder task has lower recognition rates as compared to that of Dup 1. PCA based algorithms perform generally better for both Dup 1 and Dup 2, as compared to LPP based algorithms. The best performing algorithm is A2DPCA-Cos, with 25% retained basis vectors, for both Dup 1 and Dup 2 sets.

#### 1.4. Overall

PCA-MahCos and A2DPCA-Cos are generally the best performers on the FERET database as they each achieve the top recognition rates in three out of four of the face recognition tasks. They perform well on YALE and ORL database too, but the top recognition rates are achieved by 2DPCA-Euc on ORL and 2D^2^PCA-MahCos on YALE.

### 2. Based on Facial Databases

For FERET, the best algorithms that perform equally well on all probe sets are PCA-MahCos and A2DPCA-Cos. For YALE, the best performing algorithm is 2D^2^PCA-MahCos. PCA-MahCos and A2DPCA-Cos along with 2DPCA-Euc are close too. ORL images include slight pose variations and here the best performing algorithm is 2DPCA-Euc. Other algorithms close in performance are 2D^2^PCA-Euc and A2DPCA-Euc. The algorithms performing the best on average over all databases are A2DPCA-Cos and PCA-MahCos.

### 3. Based on Distance Metrics

Though variants of Mahalanobis distance metric did not work well with 1D LPP on all three databases, yet they perform well with all PCA based algorithms and 2D LPP for all face recognition tasks on all databases. The need for experimenting with variants of Mahalanobis distance metrics was pointed out in [18]. Euclidean distance metric performs satisfactorily on average with all two dimensional PCA based algorithms, followed by Cosine distance metric, on all face recognition tasks over all databases. MahCos is the best performing distance metric with PCA on average over all face recognition tasks and databases, a result similar to that of [27] and [28].

It is worth noting that the Euclidean distance metric works well against the expression task which actually leads to the local geometrical distortions in a facial image. On the other hand, Cosine distance metric which is close to the correlation of image vectors, works well against illumination changes which are non-geometrical distortions. This general trend is evident from the results in table 4 against the facial tasks fafc (illumination) and fafb (expression). As the local geometrical distortion such as change in expression effects only a small portion of a facial image, only a few components of image vector show significant variations among genuine candidates also. While the non-geometrical distortion such as change in illumination affects the major portion of an image, therefore maximum components of image vectors show a consistent difference. The Euclidean distance handles larger variations in fewer components better as compared to correlation therefore it shows generally better results in expression tasks. Cosine on the other hand is more suitable to handle illumination variations which cause consistent change. Against the aging tasks (dup1, dup2) the results show mixed trends as evident from table 4 due to the fact that such task incorporates both the local geometrical and non-geometrical changes.

### 4. Based on Algorithms

It should be noted that there is quite a lot of variations in the performance of different algorithms and thus in the performance ranking for different type of datasets. 2D^2^PCA generally gives the highest recognition rates on both YALE and ORL database as well as for the expression test set on FERET. PCA recognition rates are highest for FERET database. A2DPCA is on average the best algorithm over all the three databases. The reason is, because this algorithm works along the rows of images. All the images of the three databases have more rows than columns, therefore this algorithm had chance to retain more information as compared to 2DPCA which works along columns. For the same number of retained basis vectors, A2DPCA consumes lesser testing time as compared to 2DPCA, because length of rows is lesser than the length of columns. To conclude, PCA based algorithms perform the best overall on all the three databases, though 2DPCA based algorithms give better recognition rates than PCA on average but with bigger template sizes. Another thing worth noting from table 4 and 5 is that all the 2DPCA based algorithms give maximum recognition rate (shaded values) for almost the same percentage of retained basis vectors. An important observation is that 2DLPP outperforms 1DLPP for almost every face recognition task on all the three databases.

### 5. Based on Memory and Computational Complexities

The sizes of covariance matrix, projection matrix, templates and the time and memory complexity of each algorithm are summarized in table 1. The dimensions *m*,*n,N,M* and *d* have been already defined in section 2. From the matrix dimensions section of the table, one can clearly understand the dimensions of the output of training and projection phase. Based on these dimensions, the algorithm complexity can easily be understood.

The training time complexity depends upon both the size of the covariance matrices and the number of retained basis vectors. Therefore for PCA it is O(m^2^n^2^d) and for 2DPCA it is O(n^2^d) due to a smaller covariance matrix. The A2DPCA has O(m^2^d) because it works along the columns and for 2D^2^PCA it is O(n^2^d_1_+ m^2^d_2_) because it has to calculate two covariance matrices, one along rows and other along columns. For LPP and 2DLPP an extra cost O(mnN^2^) to construct the adjacency matrix *S* is bared.

The testing time is calculated by the number of tests to perform and the time complexity for each test. This time also reflects the computational complexity during recognition which is very critical especially for identification systems. This turns out to be O(MN) for the number of tests, and time complexity for each test is O(d) for one dimensional algorithms and O(md) for two dimensional algorithms. So PCA and LPP have the time complexity of O(MNd), 2DPCA and 2DLPP have O(mMNd), A2DPCA has O(nMNd) and 2D^2^PCA has O(d_2_MNd_1_).

The memory cost depends on the size of the covariance matrices. Therefore for PCA and LPP it is O(m^2^n^2^), for 2DPCA and 2DLPP it is O(n^2^) and for A2DPCA it is O(m^2^). The 2D^2^PCA algorithm has a memory cost O(m^2^+ n^2^) due to the fact that it calculates two Eigen equations.

To summarize the above discussion, it is obvious that PCA variants are computationally efficient as compared to LPP variants. In an identification system the training and projection is usually done offline, while the distance calculation and recognition is done online, mostly real time, which has critical timing constraints. The above analysis shows that the training time and memory space complexity for 1D PCA, which generally demonstrates better recognition rates, is higher due to bigger covariance matrix. However it is very efficient at matching stage due to smaller template size and thus suitable for identification systems. On the other hand A2DPCA is efficient during training due to smaller covariance matrix but has a bigger template size and needs more online processing time during recognition as compared to PCA. 2D^2^PCA on average has a comparatively smaller template size and it is also efficient during matching, therefore it is the most efficient in both respects among the two dimensional PCA algorithms.

### 6. Based on Comparison to Previous Work

For comparing the results of this study, similar studies which have used one or more of the algorithm and distance metric combinations are considered here. Variation of results as compared to previous studies can be attributed to different pre-processing technique and the standard testing methodology not used by most of these studies. But as this study is an independent comparative analysis, it serves the purpose.

Regarding FERET evaluation methodology tasks, we found that the FAFB task is the easiest with highest recognition rates which is consistent with [8] [18] and FAFC is comparatively the hardest task with lowest recognition rates (on average) which is consistent with [12]. But no concrete claim could be made about either FAFC or DUP2 to be the hardest task based upon the recognition rates against them.

PCA with variants of Mahalanobis based distance metrics is experimented as more investigation was recommended by [18] and they perform very well on all the three facial databases which is consistent to [28] and [11]. 2DPCA-Euc is better than PCA-Euc on both ORL and YALE databases and similar trends hold for FERET too, which is consistent with [21]. But in this study, 2DPCA wasn’t compared with PCA using other distance metrics and in our study it is found that PCA-MahCos does equate and even surpass 2DPCA-Euc’s performance in some cases.

While using Euclidean as a distance metric, the recognition rates of all the two dimensional PCA algorithms on all three databases are pretty close to each other which is in agreement with [22]. There is also a disagreement with [22], as it states that 2D^2^PCA-Euc always performs better than both PCA-Euc and 2DPCA-Euc for lower number of retained dimensions. Our study shows that such claim holds valid against PCA-Euc only because 2DPCA-Euc is almost equal in performance over all the three databases.

Regarding the 2DLPP algorithm, our results are not in agreement with [24]. Though for some of the distance metrics, the recognition rate of 2DLPP is comparable to that of 2DPCA, but for the Euclidean distance metric, 2DLPP is clearly behind 2DPCA-Euc for all the three databases.

## Conclusion and Future Directions

The aim of this study was to independently compare and analyze the relative performance of famous subspace face recognition algorithms against the same working conditions. As mentioned in the testing methodology section, we have followed the FERET evaluations methodology which closely simulates real life scenarios. Six popular subspace face recognition algorithms were tested accompanied with four popular distance metrics.

**Figure 4:**
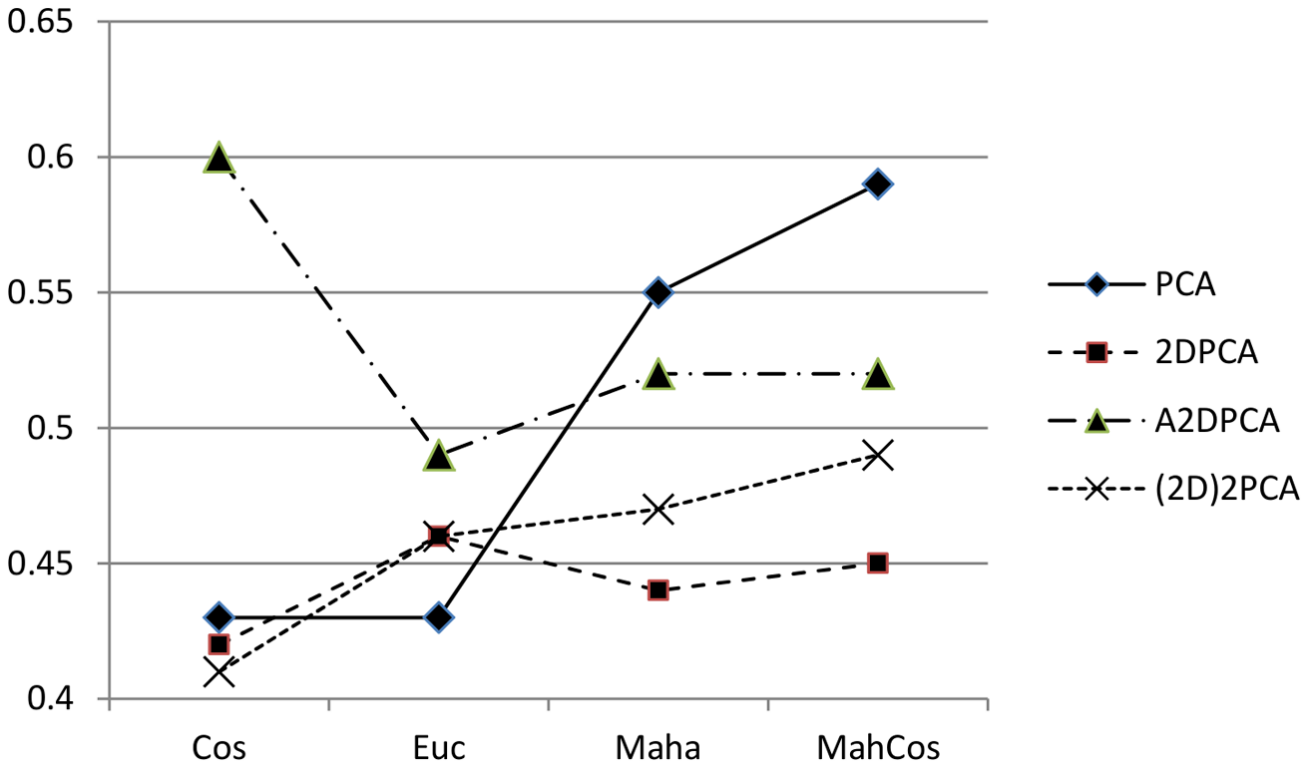
Average recognition rates of PCA based algorithms against distance metrics for FERET.

An important and novel contribution of this study is that it introduced an unbiased comparative analysis of popular subspace algorithms under equal and testing working conditions, such as same pre-processing steps, same testing criteria, same testing and training sets and also introduced the favorable performance conditions for each of these algorithms. After thorough experimentations it was shown that Algorithm 1D PCA performed best with distance metric Mahalanobis-Cosine, and 2DPCA variants and 1D LPP performed generally much better with simple Euclidean and Cosine distance metrics. Similarly 2DLPP performed much better with distance metrics Mahalanobis and Mahalanobis-Cosine. In addition to this it was shown that Cosine based distance metrics, MahCos and Cos, gave better results than Euclidean based metrics. The algorithm-metric combination of PCA-MahCos was clearly ahead in performance under difficult conditions of illumination changes. As evident from figure 4, generally for all tasks A2DPCA-Cos was found to be better than other combinations especially against aging tasks.

A thorough computational complexity analysis was also performed on the subject algorithms. It was shown that though 2D algorithms have lower complexity during training, they need more computations during recognition which is critical for identification systems. On the other hand 1D algorithms have higher computational complexity during training but generally require less computations during recognition stage.

It was also noted that the performance variations are very significant for different databases. Any algorithm alone cannot be qualified as the best performing algorithm for all the variations of a facial image. To extract the optimal performance on all facial variations, it may be necessary to combine several subspace techniques in a computationally economical unified classifier which makes a good research topic for future.

A MATLAB based evaluation platform was also constructed in result of this study which may serve as a useful tool for researchers in this field.
